# Editorial: Exploring metabolic-based host-pathogen interactions

**DOI:** 10.3389/fpls.2023.1247913

**Published:** 2023-08-21

**Authors:** Yasser Nehela, Osama Atallah, Tran Dang Xuan, Abdelnaser A. Elzaawely

**Affiliations:** ^1^Department of Agricultural Botany, Faculty of Agriculture, Tanta University, Tanta, Egypt; ^2^Department of Plant Pathology, Faculty of Agriculture, Zagazig University, Zagazig, Egypt; ^3^Department of Plant Pathology & Microbiology, Faculty of Agriculture & Life Sciences, Texas A&M University, College Station, TX, United States; ^4^Transdisciplinary Science and Engineering Program, Graduate School of Advanced Science and Engineering, Hiroshima University, Higashi-Hiroshima, Japan; ^5^Center for the Planetary Health and Innovation Science (PHIS), The International Development and Cooperation (IDEC) Institute, Hiroshima University, Higashi-Hiroshima, Japan

**Keywords:** multi-omics, metabolomics, phytopathogens, primary metabolites, secondary metabolites, hostpathogen interactions, viral disease, fungal disease

## 1 Introduction

Land plants are an important element of our ecosystem where they fulfill key human needs such as food, textiles, housing, and medicine. The growth, yield, and survival of plants are highly affected by different abiotic and biotic stresses, including phytopathogen infections. Plants are attacked by a wide range of prokaryotes, fungi, nematodes, and viruses. Upon pathogen infection, they develop a sophisticated multi-layered defensive system to protect themselves and efficiently counterattack the pathogen infections. This defensive system composite of multiple morphological, biochemical, and physiological mechanisms such as the strengthening of cell walls, generation of reactive oxygen species (ROS), activation of the innate immune system, and alteration of several metabolic pathways (Kaur et al., 2022). The metabolic changes of host plants may be cellular functions for defense reactions, or they may be triggered by pathogens to fulfill their nutritional needs. It is worth mentioning that successful pathosystems are linked with the ability of the pathogen to overcome the host’s defensive mechanisms, as well as to obtain nutrients from their host.

**Figure 1:**
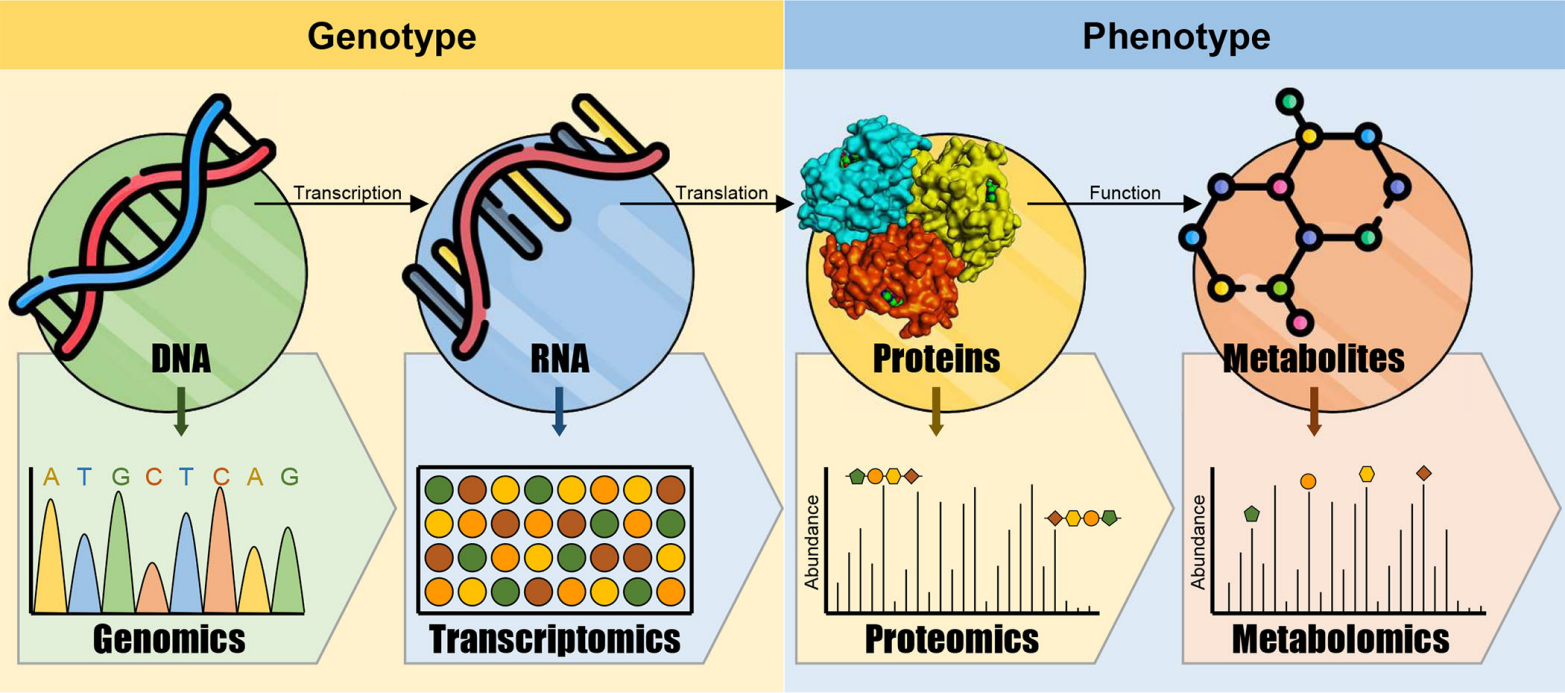
Conceptual model of multi-omics in plants. Omics data acquiesced from various cellular levels such as the genome, transcriptome, proteome, and metabolome and integrated and analyzed to better understand physiological, biological, and molecular mechanisms of host-pathogen interactions.

Generally, the pathogen-triggered metabolic changes are implicated directly or indirectly in host defensive responses. Briefly, upon the infection, the host plant reprograms its genome and transcriptome commanding changes in plant proteome and metabolome (**Figure 1**). Metabolic-based host-pathogen interactions include the accumulation of defense-related molecules, alteration of plant signaling system, and changes in primary and secondary metabolites. Moreover, metabolic homeostasis is interconnected with the innate immune system of the host plant. Furthermore, both primary and secondary metabolites play a key role to support the required cellular energy for defensive responses of the host plant (Bolton, 2009) which appears to impose a fitness cost. Consequently, it was hypothesized that the augmentation of defense-related pathways is usually compensated by the reduction of other metabolic pathways to secure a beneficial energy balance for defense (Rojas et al., 2014).

Although numerous investigations have been carried out to better understand how plants metabolically respond to various biotic stressors, only a few of them explored the metabolic-based host-pathogen interactions (Matilla, 2018). For example, our previous study showed that infection with the bacterial pathogen “*Candidatus* Liberibacter asiaticus” caused numerous metabolic changes in citrus plants. These include changes in amino acids, organic acids, and fatty acids pathways (Killiny and Nehela, 2017a), photosynthetic pigments (Killiny and Nehela, 2017b), phytohormones (Nehela et al., 2018), tricarboxylic acid cycle (TCA)-associated compounds, non-proteinogenic amino acids, and polyamines (Nehela and Killiny, 2019), the auxin-like compound melatonin (Nehela and Killiny, 2020), and *γ*-aminobutyric acid (GABA) shunt (Nehela and Killiny, 2022).

Metabolomics attempts to measure the unique chemical fingerprints and small-molecule substrates, commonly known as metabolites, intermediates, and products within a specific tissue or fluid at a specific time point (Hollywood et al., 2006; Hall, 2011). Metabolomics has become a powerful tool to better understand how plants respond to various biotic and abiotic stressors (Hall, 2011).

In the current Research Topic “*Metabolic-based host-pathogen interactions*”, four original research articles were received, that focus on (i) virus-triggered metabolites in potatoes (Manasseh et al.), (ii) resistance-associated metabolites of soybean seed to *fusarium fujikuroi* (Chang et al.), (iii) plant growth-promoting rhizobacteria (PGPR)-induced metabolites in wheat infected with *Puccinia striiformis* f. sp. *tritici* (Mashabela et al.), and (iv) identification of novel antioxidant flavonoids associated with drought tolerance in citrus (Rao et al.). Moreover, one review article that discussed the potential role(s) of *Streptomyces*-produced metabolites in the control of phytopathogens (Dow et al.) was received.

## 2 Exploring metabolic-based host-pathogen interactions

### 2.1 Role(s) of host metabolites against phytopathogens

#### 2.1.1 Viral pathogens

Leaf metabolites play a key role in host defensive responses against viral (Manasseh et al.) and fungal diseases (Chang et al.; Mashabela et al.). Manasseh et al. used untargeted metabolomics to investigate the responses of potato virus Y (PVY)-resistant cv. Premier Russet and susceptible cv. Russet Burbank to the inoculation with different PVY strains including PVY^NTN^, PVY^N-Wi^, and PVY°. Generally, more than 45 common and/or strain-specific metabolites were detected in fully expanded leaves after the extraction by a solvent containing methanol, 2-propanol, and water (5:2:2; v/v/v) and derivatization with O-methoxylamine hydrochloride dissolved in pyridine and N-methyl-N-(trimethylsilyl)-trifluoroacetamide (MSTFA) containing 1% trimethylchlorosilane (TMCS). Briefly, the GC-MS-based analysis revealed PVY infection in potatoes leads to marked changes in amino acid, energy, and fatty acid metabolism, with a focus on 20 amino acids, 11 organic acids, and 14 sugar derivatives. It is worth noting that the accumulation of L-allothreonine, mannose, and fructose was stimulated by all three strains of PVY.

In the PVY-resistant cv. Premier Russet only two differentially accumulated metabolites (DAMs) were detected under PVY^NTN^ inoculation compared to six and 13 DAMs upon the infection with PVY° and PVY^N-Wi^, respectively (Manasseh et al.). On the other hand, in the PVY-susceptible cv. Russet Burbank, PVY^NTN,^ and PVY° strains pronounced higher metabolic changes (13 and 12 DAMs, respectively), compared to the PVY^N-Wi^ strain which altered only four DAMs. Moreover, although 14 metabolic pathways occurred solely in PVY^N-Wi^-infected potato plants, a major overlap was noticed between PVY^N-Wi^ and PVY° in Premier Russet, and eight DAMs were detected specifically in PVY^N-Wi^-infected leaves, whereas DAMs of PVY^NTN^ and PVY° overlapped in Russet Burbank. Taken together, the authors suggest that five metabolites included; maltose, D-glyceric acid, L-malic acid, lysine, and oxoproline, were unique to Premier Russet, and another five metabolites included; L-allothreonine, 5-methoxytryptamine, spermidine, fructose, and aconitic acid, were cultivar-specific for Russet Burbank which could be used as potential biomarkers/indicators of PVY infection and susceptibility/resistance of potato genotypes (Manasseh et al.).

#### 2.1.2 Soil-borne fungal pathogens

In another study, Chang et al. investigated the physiological and metabolic responses of soybean seed of the moderately susceptible cultivar “Nandou 12” after soaking in spore suspension of the seed-borne phytopathogen *Fusarium fujikuroi* - isolate No. S88, the causal agent of seed decay, at 0, 3, and 5 days post-inoculation (dpi). Although both mycelial coverage percentage and disease severity index increased rapidly upon the inoculation with *F. fujikuroi*, the germination rate and the levels of soluble sugars and soluble protein of treated seeds were markedly altered over time. Moreover, the enzymatic activities of cell wall–degrading enzymes such as chitinase and *β*-1,3-glucanase of soybean seeds were promptly stimulated after the infection with *F. fujikuroi*. Collectively, these findings suggest that the seeds of the moderately susceptible cultivar “Nandou 12” might accumulate more soluble protein and activate the cell wall–degrading enzymes as early defense strategies against *F. fujikuroi*, but reduce the soluble sugar content to improve the respiration of infected seeds.

To better understand the potential role(s) of the key defense-related metabolites and their corresponding metabolic pathways during the soybean-*F. fujikuroi* interactions, the authors examined the metabolic responses of soybean seed after the extraction with 80% methanol and 0.1% formic acid and analyzed using Ultra Performance Liquid Chromatography Tandem Mass Spectrometry (UPLC-MS/MS). Metabolic profiling revealed that soybean seed metabolites were altered by *F. fujikuroi* including secondary metabolites, amino acids, carbohydrates, cofactors & vitamins, and lipid metabolism. Briefly, the metabolism of glycine, serine, threonine, and tryptophan was noticeably provoked by *F. fujikuroi*. Moreover, infection with *F. fujikuroi* significantly activated the isoflavonoid metabolites in soybean seeds. For instance, the upstream metabolites such as naringenin and liquiritigenin in the isoflavone biosynthesis pathway were rapidly accumulated at 3 dpi but diminished at 5 dpi. On the other hand, downstream metabolites such as daidzin, daidzein, genistin, and genistein decreased, while liquiritigenin, glycitein, and glycitin notably increased at 3 dpi and then rapidly decreased. Taken together, the authors suggest that the accumulation of isoflavonoid metabolites in soybean seeds might play a key role in response to *F. fujikuroi* infection by acting as a signal of early defense response.

#### 2.1.3 Obligate biotrophic fungal pathogens

In a previous study, Mashabela et al. used untargeted metabolomics to explore the metabolic profile of the rhizosphere and the above-ground tissues of susceptible and resistant wheat after the application of two PGPR; namely *Paenibacillus alvei* - T22 and *Bacillus subtilis* under neither biotic nor abiotic stress (Mashabela et al., 2022). Briefly, several metabolic pathways/groups were differentially accumulated in the rhizosphere and leaves of PGPR-treated plants including phenylpropanoids, organic acids, lipids, organoheterocyclic compounds, and benzenoids. These metabolites are implicated in chemotaxis, biocontrol, plant growth promotion, and host–pathogen interactions (Mashabela et al., 2022). In the current Research Topic, they added another crucial piece to the puzzle to evaluate the capabilities of PGPR in stimulating plant growth and inducing systemic resistance to protect wheat plants against the obligate biotrophic phytopathogen, *Puccinia striiformis* f. sp. *tritici* (*Pst*), the causal agent of stripe rust (Mashabela et al.). In addition, Mashabela et al. tried to identify signatory biomarkers of the *Pst*-susceptible Gariep cultivar against *Pst* after bio-priming with two PGPR strains namely *B. subtilis* and *P. alvei* - T22 under greenhouse conditions using untargeted Ultra-High Performance Liquid Chromatography-High-Definition Mass Spectrometry (UHPLC-HDMS).

Mashabela et al. reported that seed bio-priming using *B. subtilis* and *P. alvei* showed potential differential metabolic responses between treated and non-treated plants. These metabolic responses included but were not limited to, time-dependent reprogramming of primary metabolites (amino acids and organic acids), as well as secondary metabolites such as phenolic compounds (flavonoids, hydroxycinnamic acid, and its amides). It is worth mentioning that these metabolic responses were further reflected by a reduction in the development of disease symptoms as suggested by visual inspection of the plant phenotypes. PGPR-treated plants develop chlorosis and necrosis at the site of infection with *Pst*, and a reduced infection rate is recognized by developing smaller and fewer spores compared to the non-primed Gariep plants. These findings correspond to the observations from the *Pst*-resistant wheat cultivar Koonap in their previous study(Mashabela et al., 2022) which suggest that bio-priming using *B. subtilis* and *P. alvei* alters the metabolic profile linked to induced resistance against *Pst* in the treated plants. The current study of Mashabela et al. is an important contribution to the field of metabolomics-based semi-quantitative and qualitative analyses to clarify wheat-*Pst* interactions and to identify primary and secondary metabolic biomarkers associated with these interactions.

### 2.2 Role(s) of host metabolites against abiotic stress

In the current Research Topic, Rao et al. aimed to identify novel antioxidant flavonoids associated with drought tolerance in the leaves of three citrus varieties including the drought-tolerant sour orange (*Citrus aurantium*), and two drought-sensitive varieties ‘Majia you’ pummelo (*Citrus maxima*), and lemon (*Citrus* × *limon*) grown under controlled conditions. Flavonoids were extracted using 70% methanol and analyzed using UPLC-MS/MS. Generally, Drought stress differentially reprogramed the profile of approximately 37 flavonoids of the three studied citrus varieties including 12 flavones, ten flavonols, six flavanones, five isoflavanones, and one chalcone, flavanol, flavanonol, and flavone glycoside. In the drought-tolerant variety sour orange, vitexin, neohesperidin, cynaroside, hyperoside, genistin, kaempferol 3-neohesperidoside, eriocitrin, and luteolin were notably accumulated, particularly after 18 days of drought stress, however, these compounds were slightly diminished in lemon or even did not change in response to the same stress. For instance, the flavonol kaempferol 3-neohesperidoside was 17 folds higher in the drought-tolerant variety sour orange than the drought-sensitive variety lemon. Likewise, the flavanone neohesperidin was higher in the leaves of sour orange than in drought-sensitive varieties ‘Majia you’ pummelo, and lemon (about 1407 and 37 folds, respectively) after 18 days of drought stress.

Moreover, cluster analysis showed that eight flavonoids were uniquely higher in the leaves of sour orange after 18 days of drought stress including cynaroside, 2’-hydroxygenistein, 2’-hydroxydaidzein, calycosin, chrysin, neohesperidin, kaempferol 3-neohesperidoside, and vitexin. However, only three flavones included; Apigenin, Astragalin, and Limocitrin, and one chalcone (Naringenin chalcone) were higher in ‘Majia you’ pummelo than in other varieties. These compounds might be used as biomarkers for drought tolerance in citrus varieties. It is worth mentioning that the metabolic reprogramming of citrus flavonoids was associated with higher total flavonoids contents and antioxidant activity, as well as upregulation of most flavonoid biosynthesis genes included; *PAL*, *CHI*, *FLS*, *GT1*, *F3H*, *F3’M*, *C4H*, *4CL*, *FLS*, *FG2*, *FG3*, and *CYP81E1*. Collectively, these findings suggest that while the drought-tolerant variety sour orange was able to accumulate higher levels of antioxidant-associated flavonoids with augmented antioxidant activity to detoxify the harmful effects of ROS formed during extended drought stress, drought-sensitive varieties ‘Majia you’ pummelo, and lemon were unsuccessful to biosynthesize antioxidant-associated flavonoids to survive the prolonged drought stress.

### 2.3 Role(s) of microbial metabolites against phytopathogen

In the current Research Topic, Dow et al. well-reviewed the potential role(s) of *Streptomyces*-specialized metabolites in the biological control of phytopathogens and how, what, when, and where *Streptomyces*-specialized metabolites mediate the host-pathogen interactions as well as host-associated microbial communities. The authors suggested that the plant symbionts *Streptomyces* can efficiently inhibit numerous phytopathogens *via* direct antimicrobial activity and substantially induce plant defense *via* indirect biosynthetic pathways. In the beginning, the author listed a non-exhaustive list of well-characterized *Streptomyces*-specialized metabolites, which have been investigated *in planta*, and the situation of registering these metabolites as commercial products under different trademarks. Although at least 150,000 *Streptomyces*-specialized metabolites are currently known (Lacey and Rutledge, 2022), the majority of these metabolites were not examined *in planta* but only tested for their antimicrobial activity using *in vitro* assays and/or cell-free fermentation extracts.

Therefore, the authors well-discussed the challenges of traditional *in vitro* approaches for microbial biological control agents (MBCA) and the discovery of their specialized metabolites. These challenges include but are not limited to, (i) changes in the nutrient medium which might cause alteration in the produced metabolites with unreliable efficacy against targeted phytopathogens; (ii) poor understanding of the ecological signals that trigger the production of specialized metabolites; and (iii) the lack of translation from *in vitro* screens to *in planta* bioactivity which leads the identification of promising biocontrol agents, but significantly restricts their field application. Additionally, the authors discussed some of the customized *in vitro* techniques used for the production and testing the *Streptomyces*-specialized metabolites, as well as some of *in planta* methodologies for biocontrol and discovery of specialized metabolites. Finally, they discussed the potential utilization of multi-omics approaches and biosensors as direct or indirect reporters for the discovery, deployment, and manipulation of *Streptomyces*-specialized metabolites for plant protection.

## 3 Conclusion

**Figure 2:**
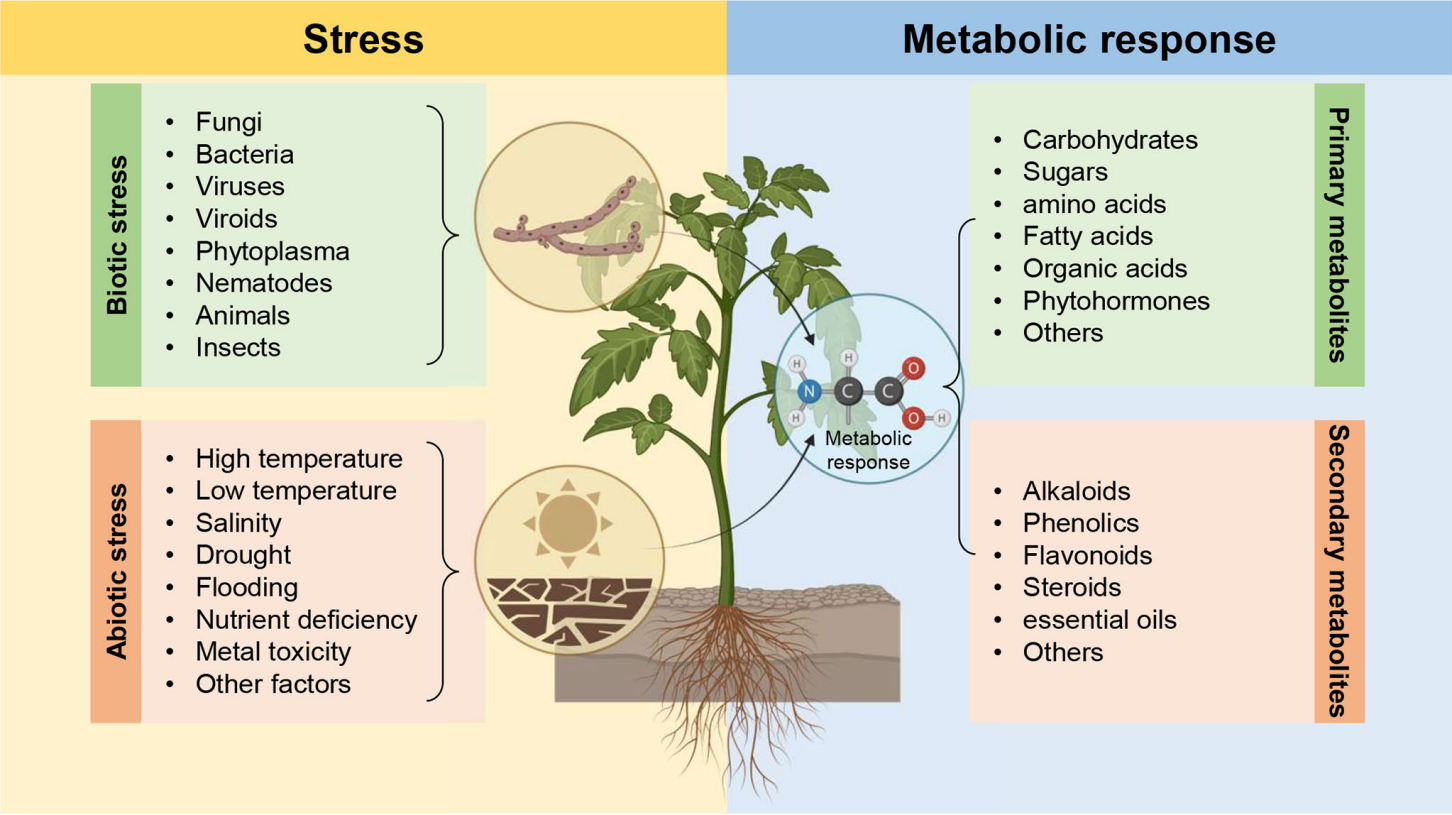
Plants rely on a sophisticated multi-layered metabolic defensive system to protect themselves and efficiently counterattack the harmful effects of biotic and abiotic stress.

Plants rely on a sophisticated multi-layered metabolic defensive system to protect themselves and efficiently counterattack the harmful effects of biotic and abiotic stress (**Figure 2**). Large-scale metabolomic analysis of different plant species under biotic stress generally, and pathogen infection particularly, revealed that pathogen infection altered the abundances of several primary metabolites such as carboxylic compounds, glycolysis, pentose phosphate, and shikimate pathways, as well as secondary metabolites such as terpenoid/isoprenoid, phenolic compounds, and nitrogen/sulfur-containing compounds. Metabolic changes play a central role in determining the outcome of host-pathogen interactions which is usually associated with the availability and flux of nutrients in the infected plant. In other words, these metabolic changes could be implicated directly or indirectly in host-pathogen interactions to provide sufficient strength and rigidity to ease the harmful effects caused by phytopathogens. On the other hand, phytopathogens themselves may employ several metabolites to be used as virulence factors or to interfere with host immunity. In this context, a better understanding of metabolic reprogramming and a comprehensive perspective of how metabolic networks are regulated during the host-pathogen interaction is necessary to elucidate the complex interactions between the plant and phytopathogenic microbes. Finally, we believe that the current Research Topic “*Metabolic-based host-pathogen interactions*” highlights interesting avenues for the potential role(s) of host/microbe primary and secondary metabolites against phytopathogens, as well as abiotic stress and to understand better host-pathogen interactions at the metabolic level.

## Author contributions

YN conceptualized the idea and wrote the draft manuscript. All authors have made a considerable intellectual contribution to the work and revised the final version of the manuscript. All authors have read and agreed to the published version of the manuscript.
